# Systemic immune profiling in hepatocellular carcinoma: navigating etiological heterogeneity and selection bias

**DOI:** 10.1136/jitc-2026-015777

**Published:** 2026-07-01

**Authors:** Rihui Chen, Xiaochuan Yang

**Affiliations:** 1 Haikou Municipal People’s Hospital, Haikou, Hainan, China

**Keywords:** Hepatocellular Carcinoma, Immunotherapy, Partial response, Immune Checkpoint Inhibitor, T cell

## Abstract

This correspondence provides a methodological commentary on the recent study by Nishio *et al*, which used single-cell RNA sequencing (CITE-seq) to identify circulating immune biomarkers for combination immunotherapies in advanced hepatocellular carcinoma (HCC). While the original study offers high-resolution insights into systemic immunity, we identify three critical areas that warrant further consideration to ensure the clinical validity of the proposed biomarkers. First, we discuss how etiological heterogeneity (viral vs non-viral HCC) may confound regimen-specific immune signatures. Second, we highlight the potential for “selection bias” and statistical inflation of effect sizes resulting from the balanced 1:1 responder to non-responder study design, as well as the inherent mathematical artifacts in compositional single-cell data analysis. Third, we question the biological applicability of modeling stable ligand-receptor interactions (CellChat) within the high-flow environment of peripheral circulation. We conclude that future validation in unselected, prospective cohorts is essential to confirm whether these circulating immune subsets can serve as robust clinical predictors in real-world settings.

We read with great interest the study by Nishio *et al*, which used single-cell RNA sequencing (CITE-seq) to uncover divergent systemic immune mechanisms and predictive biomarkers for combination immunotherapies in advanced hepatocellular carcinoma (HCC).^1^ The authors elegantly demonstrated how different regimens, namely atezolizumab/bevacizumab (Atez/Bev) and durvalumab/tremelimumab (Dur/Tre), remodel the circulating immune landscape. While these findings provide high-resolution insights into systemic immunity, we believe several methodological nuances regarding study design and statistical inference merit further discussion.

First, the authors identified distinct baseline immune states between viral and non-viral HCC, specifically noting that non-viral HCC exhibits CD8+T cells with heightened cytotoxic and memory-associated signatures. However, in the subsequent comparative analysis between Atez/Bev and Dur/Tre responders, it remains unclear whether the treatment cohorts were balanced for etiology. Given the profound impact of background liver disease on systemic T-cell fitness,^2^ the reported regimen-specific mechanisms might partially reflect baseline etiological differences rather than the direct effect of the specific therapeutic agents.

Second, and perhaps most critically, we have concerns regarding the “selection bias” inherent in the predictive biomarker assessment. The investigators selected a balanced number of responders (R) and non-responders (NR) for single-cell analysis (eg, five R vs five NR for Atez/Bev). While this “1:1 case-control” design is efficient for identifying differentially expressed genes, it creates an artificial prevalence of responders that does not reflect the real-world clinical setting, where objective response rates for these combinations typically range from 20% to 30%.

Specifically, the authors report that pretreatment frequencies of CCR7+TCF7+ naïve CD4+ T cells and PRKCH+ NK cells predict response to Atez/Bev. However, the statistical power and predictive value of these subsets are highly sensitive to the prevalence of the outcome in the study population. In an artificially balanced cohort, the effect size of frequency-based markers can be significantly exaggerated.^3^ Without validation in an unselected, prospective cohort that maintains the natural distribution of treatment response, it is premature to conclude that these circulating frequencies can serve as reliable clinical predictors. Furthermore, frequency-based analysis of single-cell data are inherently compositional; an increase in one subset (eg, monocytes in non-responders) mathematically forces a relative decrease in others (eg, T cells), potentially leading to “pseudopredictors” that reflect mathematical artifacts rather than independent biological signals.

Third, the biological interpretation of intercellular communication in the circulation warrants caution. The study uses CellChat to model monocyte-natural killer and monocyte-T cell interactions based on PBMC transcriptomes. While these computational models identify potential ligand-receptor axes, the high-flow environment of the peripheral blood suggests that these cells may lack the stable spatial proximity required for the inferred physical interactions.^4^ Therefore, projecting mechanisms inferred from circulating cells directly onto the primary tumor’s therapeutic response involves a significant logical leap that requires further validation in the tumor microenvironment.

In conclusion, while Nishio *et al* provide a valuable framework for understanding systemic immune dynamics in HCC, the interplay between etiology and treatment regimen, along with the impact of artificial responder prevalence on biomarker discovery, should be carefully considered in future validation studies.
